# Antifibrotic Effects of Roscovitine in Normal and Scleroderma Fibroblasts

**DOI:** 10.1371/journal.pone.0048560

**Published:** 2012-11-20

**Authors:** Richard A. Steinman, Andria Rasile Robinson, Carol A. Feghali-Bostwick

**Affiliations:** 1 Department of Medicine, University of Pittsburgh School of Medicine, Pittsburgh, Pennsylvania, United States of America; 2 Department of Pharmacology, University of Pittsburgh School of Medicine, Pittsburgh, Pennsylvania, United States of America; 3 Department of Pathology, University of Pittsburgh School of Medicine, Pittsburgh, Pennsylvania, United States of America; H. Lee Moffitt Cancer Center & Research Institute, United States of America

## Abstract

Heightened production of collagen and other matrix proteins underlies the fibrotic phenotype of systemic sclerosis (SSc). Roscovitine is an inhibitor of cyclin-dependent kinases that promote cell cycling (CDK1, 2), neuronal development (CDK5) and control transcription (CDK7,9). In an *in vivo* glomerulonephritis model, roscovitine treatment decreased mesangial cell proliferation and matrix proteins [1]. We investigated whether roscovitine could regulate fibrotic protein production directly rather than through cell cycling. Our investigations revealed that roscovitine coordinately inhibited the expression of collagen, fibronectin, and connective tissue growth factor (CTGF) in normal and SSc fibroblasts. This effect occurred on a transcriptional basis and did not result from roscovitine-mediated cell cycle inhibition. Roscovitine-mediated suppression of matrix proteins could not be reversed by the exogenous profibrotic cytokines TGF-β or IL-6. To our knowledge, we are the first to report that roscovitine modulates matrix protein transcription. Roscovitine may thus be a viable treatment option for SSc and other fibrosing diseases.

## Introduction

Systemic sclerosis (SSc), or scleroderma, is a disease with a prevalence of approximately 240 per million characterized by cutaneous and systemic fibrosis leading to significant morbidity and mortality [2]. SSc has been linked to dysregulation of immune responses and of cytokines along with upregulation of matrix proteins such as collagen and fibronectin [3]. Cytokines linked to the fibrotic phenotype include TGF-β [4], [5], [6], connective tissue growth factor (CTGF), and interleukin-6 (IL-6) [7], [8]. While antibodies directed against these targets are being tested in clinical trials, an initial trial of TGF-β antibody did not show efficacy in SSc [9] [5], while CTGF antibody trials in fibrosis are just beginning (NCT01217632). IL-6 antibody, although in trials against cancer, has not been published in use against SSc aside from an exploratory case report [10]. Emerging strategies target downstream signaling molecules in pathways transduced by pro-fibrotic cytokines [11]. However, the need for novel therapeutic approaches for SSc remains.

We investigated whether modulating cyclin-dependent kinase (CDK) activity could alter matrix protein and cytokine production by SSc fibroblasts. CDK activity is essential for cell cycle progression. In addition, CDKs can modulate gene expression independent of their cell cycle effects [12], [13], [14]. Targeting CDK activity in SSc could inhibit fibroblast proliferation or could directly inhibit matrix production. These effects are likely to be distinct since we previously reported that proliferation and collagen production were not coupled in SSc fibroblasts and that an increase in collagen production was not due to increased fibroblast proliferation [15].

Roscovitine is a purine analogue that inhibits CDKs with a high specificity for CDK1, 2, 5, 7, and 9 [16], [17], [18]. In addition to inhibiting CDKs, roscovitine has been reported to activate the ERK1/2 and HIPK2 kinases [19], [20]. Roscovitine has been used alone in oral form (roscovitine R-isomer, seliciclib) or in combination with chemotherapeutic agents in cancer clinical trials, and has shown preclinical activity against diverse cancers [21], [22]. In a mouse model of mesangial proliferative glomerulonephritis, roscovitine prevented extracellular matrix production and renal disease [1]; this effect was thought to be secondary to the inhibition of cellular proliferation by roscovitine. In normal human fibroblasts, roscovitine causes cell cycle arrest by inhibiting CDK2 [23]. Additionally, roscovitine inhibits CDK7 that phosphorylates and activates CDK2. By inhibiting CDK1 and CDK2, roscovitine prevents cell cycle progression.

Roscovitine inhibition of the transcriptional CDKs (CDK7 and CDK9) has an impact on gene expression. In contrast to flavopiridol, a CDK-inhibitor that blocks global transcription, roscovitine selectively alters transcription, causing upregulation or suppression of gene expression [14], [24], [25]. No alteration of matrix gene transcription by roscovitine has been reported to date.

Our experiments revealed that roscovitine coordinately inhibited the expression of collagen, fibronectin and CTGF in normal and SSc fibroblasts. This inhibition occurred at the mRNA level rather than as an epiphenomenon of cell cycle inhibition, and could not be reversed by exogenous TGF-β or IL-6.

## Results

### Inhibition of collagen and fibronectin expression by roscovitine

**Figure 1:**
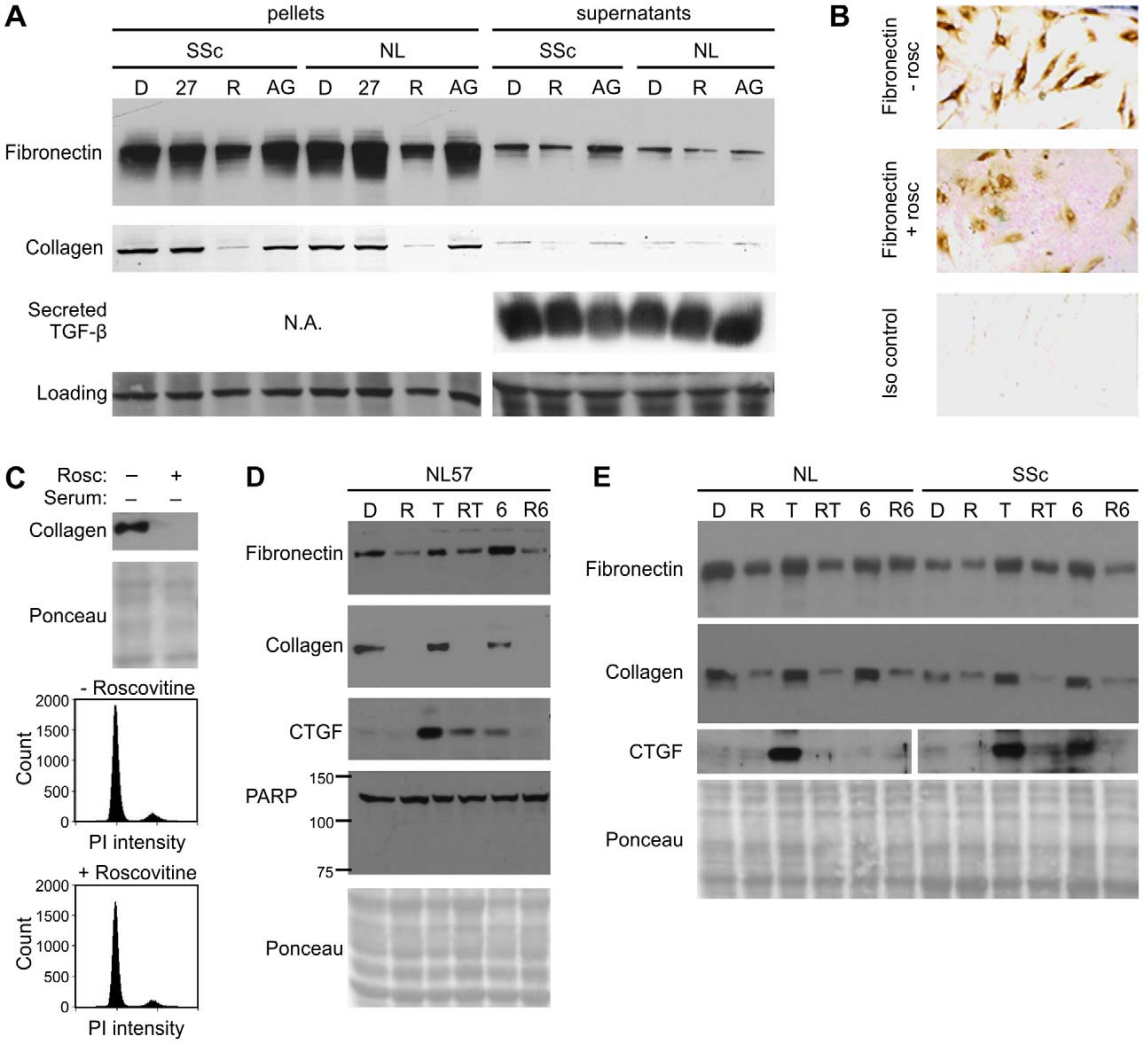
Inhibition of collagen and fibronectin expression by roscovitine in SSc and in normal fibroblasts. **A.** Passage 4 primary fibroblasts from a scleroderma patient (SSc) or her unaffected twin (NL) that had been confluent for 2 days were exposed for 36 hours to DMSO vehicle (D), roscovitine (R, 12.5 µg/mL), AG-490 (AG, 25 µM) or TAT-p27 (27, 150 nM) as shown. Equal amounts of protein are present in all lanes from the cell pellets as indicated by a non-specific cross-reactive band. The effects of these agents on matrix protein levels in the cell pellets and in cell supernatants are shown as indicated. Equal amounts of protein are present in all lanes of the supernatants as shown by a different non-specific cross-reactive band present only in the supernatant. N.A., Not applicable. **B.** Immunohistochemical staining for fibronectin production by normal primary fibroblasts cultured in the absence (−) or presence (+) of 12.5 µg/mL of roscovitine for 36 hours. **C.** Levels of collagen in fibroblasts growth arrested by culture for 9 days in 0.2% serum and subsequently exposed for 24 hours to DMSO vehicle (−) or to roscovitine (+) as indicated. Ponceau as a loading control is included. The cell cycle profiles of these fibroblasts stained with propidium iodide (PI) is also shown. **D.** Roscovitine decreases fibronectin in primary fibroblasts (NL57) exposed to exogenous TGF-β or IL-6. Roscovitine also blocked TGF-β or IL-6 induction of connective tissue growth factor (CTGF). Cells treated for 23 hours with DMSO vehicle (D), 6.25 µg/mL roscovitine (R), 10 ng/mL TGF-β (T), or 50 ng/mL IL-6 (6). PARP band at 116 kD is also shown; no 89 kD cleaved PARP band (a marker of apoptosis) was detected. Total protein loading detected by Ponceau staining was equivalent in all lanes. **E.** Primary fibroblasts from a scleroderma patient (SSc) or an unaffected twin (NL) treated as described in panel D. CTGF, run on a parallel gel had marker lane (cut out, replaced by spacer) separating NL and SSc fibroblasts.

We determined the effects of CDK inhibitors on expression of collagen and fibronectin in confluent SSc and normal fibroblasts. Two inhibitors were tested, the natural CDK-inhibitor p27Kip1 (p27), delivered as a TAT-fusion construct [26], and roscovitine. In addition, the JAK2 kinase inhibitor AG490 was tested. AG490 does not overlap with roscovitine targets but has been reported to block glucose-mediated upregulation of TGF-β and fibronectin in mesangial cells [27]. As shown in Figure 1A, roscovitine decreased both collagen and fibronectin expression in fibroblasts. A decrease in both intracellular (pellet) and secreted (supernatant) collagen type I and fibronectin was seen. Primary normal (unaffected twin) and SSc (affected twin) dermal fibroblasts derived from an identical twin pair exhibited similar responses to roscovitine. Comparable responses were seen for primary fibroblasts from two other identical twin pairs discordant for SSc (data not shown and Figure 1E). Extracellular TGF-β is the major fibrosis-associated cytokine. We measured secreted TGF-β in order to determine whether roscovitine blocked TGF-β secretion by the fibroblasts, thereby suppressing matrix proteins. As shown in Figure 1A, roscovitine could inhibit matrix proteins even in the ongoing presence of TGF-β production and secretion. Notably, the JAK2 inhibitor AG490 had no effect on matrix protein levels (quantitation shown for all blots, Table S1 in File S1). Immunohistochemical staining of normal primary fibroblasts confirmed a decrease of fibronectin expression after treatment with roscovitine (Figure 1B).

In the experiments represented in Figure 1, we used fibroblasts that had been confluent for two days to focus on the transcriptional effects of roscovitine rather than changes in protein levels simply resulting from cell cycle arrest. Figure 1C demonstrates that even with fibroblasts growth arrested in 0.2% serum for 9 days prior to overnight roscovitine treatment, collagen expression was decreased by roscovitine. Roscovitine decreased collagen by over 95% with minimal effect on the cell cycle profile (roscovitine vs. DMSO: G1/G0, 89% vs 87%; S, 2% vs 4%, G2/M 10% vs. 10%). Moreover, the inhibitory effects of roscovitine on collagen and fibronectin expression were not duplicated by direct protein transduction of p27, an inhibitor of CDK2 and CDK4 (Figure 1A), despite immunofluorescent and western blot evidence of successful p27 transduction (Figure S1 in File S1). The association of p27 (as opposed to the p21 cdk-inhibitor) with growth arrest was supported by its upregulation during confluence-mediated cell cycle exit (Figure S2 in File S1).

Roscovitine blocked TGF-β mediated upregulation of CTGF (Figure 1D). Moreover, suppression of fibronectin by roscovitine was not reversed by addition of exogenous TGF-β or IL-6 to immortalized fibroblasts (Figure 1D). There was no evidence based on cell counting (not shown) that roscovitine induced cell death; moreover, the level of PARP protein was unchanged, with no evidence that roscovitine caused PARP cleavage (no cleavage band or decreased full-length PARP, Figure 1D). This makes it unlikely that roscovitine was inducing fibroblast apoptosis. In addition, roscovitine blocked cytokine-mediated upregulation of CTGF in primary fibroblasts from an SSc patient and an unaffected twin. These data indicate a general effect of roscovitine on fibrogenic signaling in fibroblasts independent of disease.

### Roscovitine upregulates phospho-STAT3 and does not prevent TGF-β signaling

**Figure 2:**
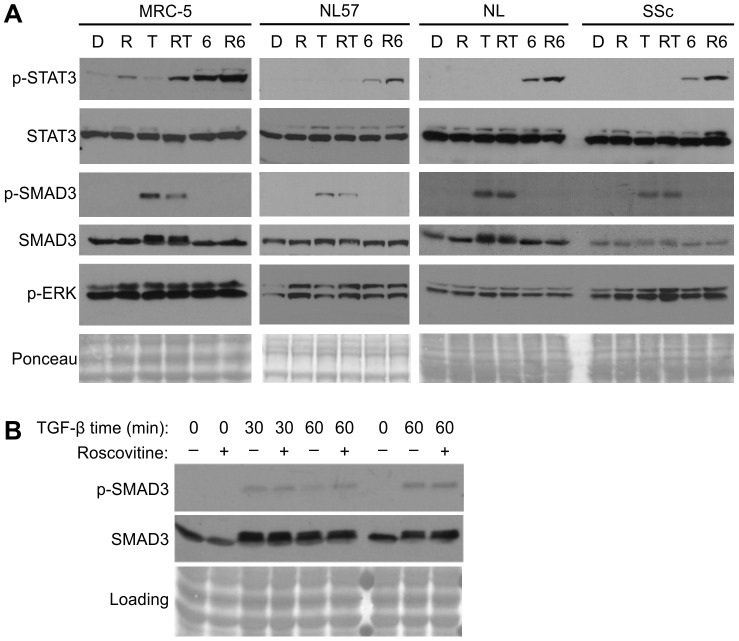
Roscovitine enhances STAT3 activation and does not block TGF-β signaling. **A.** MRC-5 and NL57 human fibroblasts, as well as primary fibroblasts from a scleroderma patient (SSc) or an unaffected twin (NL) were incubated overnight in serum-free medium and stimulated for 45 minutes with DMSO vehicle (D), roscovitine (R, 6.25 µg/mL), TGF-β (T, 10 ng/mL), or IL-6 (6, 50 ng/mL) alone or in combination as indicated. Cells were directly lysed, and proteins fractionated and blotted for phosphorylated ERK (p-ERK), STAT3 (p-STAT3), or SMAD3 (p-SMAD3) and in parallel blots for total STAT3 or SMAD3. **B.** SMAD3 activation in serum-starved MRC-5 cells exposed to TGF-β without (−) or with (+) roscovitine for up to 60 minutes as indicated in 2 parallel experiments.

We measured downstream phosphorylation events indicative of TGF-β and of IL-6 signaling in the presence and absence of roscovitine in normal human fetal lung fibroblasts (MRC-5), as well as primary adult normal and SSc dermal fibroblasts. Treatment of serum-starved fibroblasts for 45 minutes with IL-6 activated STAT3, as expected in the fibroblast cell line MRC-5 (Figure 2A). Notably, roscovitine increased STAT3 Tyr705-phosphorylation, activating both basal expression of STAT3 and increasing phospho-STAT3 in IL-6-exposed cells. Similarly, in normal primary lung fibroblasts (NL57) as well as unaffected (NL) and affected (SSc) twin dermal fibroblasts, STAT3 phosphorylation induced by IL6 was enhanced by roscovitine.

Roscovitine did not block TGF-β downstream signaling, as manifested by Smad3 phosphorylation in fibroblasts exposed to roscovitine just prior to TGF-β (Figure 2). Although a modest decrease in pSMAD3 was associated with roscovitine in some instances, roscovitine did not prevent pSMAD3 activation by TGF-β, and in fact total SMAD3 was usually shifted to a slower migrating form with TGF-β whether roscovitine was present of not. In order to confirm the magnitude of SMAD3 phosphorylation under stimulatory conditions, a timecourse after TGF-β with or without roscovitine was performed and confimed the lack of effect of roscovitine on this pathway. Interference with pSMAD3 is unlikely to contribute signficantly to the antifibrotic effect of roscovitine.

TGF-β is capable of activating non-SMAD transduction pathways, including the MAPK/ERK pathway, to activate transcription (review, [28]), however roscovitine did not alter ERK phosphorylation (Figure 2).

### Roscovitine inhibits fibronectin, collagen and CTGF transcription

**Figure 3:**
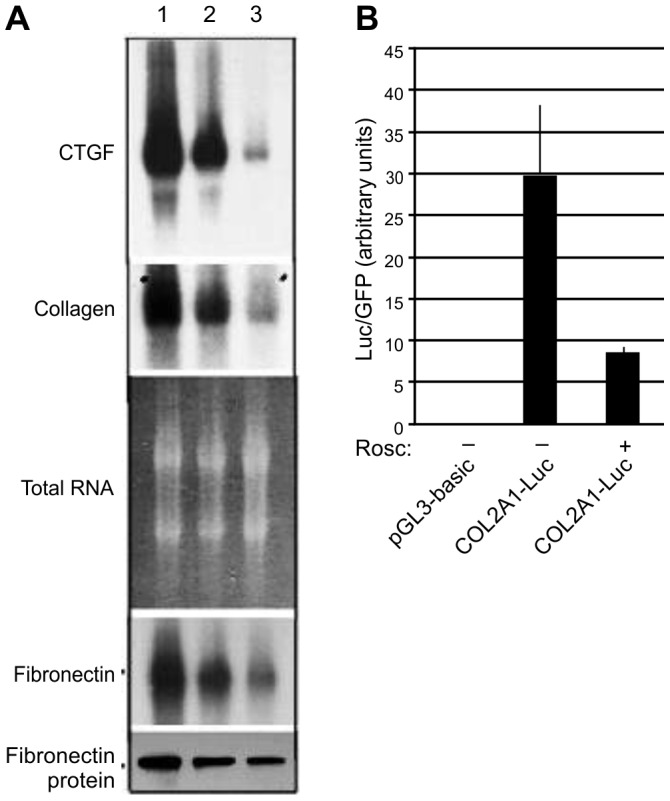
Roscovitine decreases transcription of fibrogenic mediators. **A**. Roscovitine decreases mRNA expression of *CTGF*, collagen and fibronectin. RNA from confluent primary fibroblasts incubated for 36 hours with DMSO vehicle (1), 6.25 µg/mL roscovitine (2), or 12.5 µg/mL roscovitine (3) was blotted and probed for *CTGF*, collagen, and fibronectin mRNA. Equivalent loading is shown by ethidium bromide staining for total RNA. Fibronectin protein level in these cells is shown below. **B**. Decreased transcription driven by the COL2A1 promoter in the absence or presence of 6.25 µg/mL roscovitine (Rosc). Values average three independent transfections of 3T3 cells; representative of experiments repeated 3 times. Luciferase activity is normalized to GFP protein encoded by co-transfected plasmid. *, *p* = 0.01.

To determine whether the observed decrease in collagen and fibronectin occurred at the level of gene expression, Northern blotting of primary fibroblasts in the presence and absence of roscovitine was performed. As shown in Figure 3A, roscovitine decreased mRNA levels for *CTGF*, collagen and fibronectin. The decrease in fibronectin message and protein was dose dependent. Transient transfection experiments in 3T3 cells demonstrated that roscovitine could also repress transcription driven by a collagen promoter (Figure 3B).

### Inhibition of IL-6 production

Having established that roscovitine downmodulated expression of CTGF, collagen, and fibronectin, we were interested in studying its effect on IL-6 signaling in normal and SSc fibroblasts. The production of IL-6 is markedly higher in SSc fibroblasts than in control fibroblasts [15], [29]. A downstream mediator of TGF-β [30] and/or IL-1a [31] signaling, IL-6 is a potent inducer of pro-collagen and fibronectin transcription [32], [33], [34]. The addition of roscovitine led to a dose-dependent decrease in IL-6 production as determined by ELISA (Table 1). It is notable that following roscovitine exposure, IL-6 production by the SSc fibroblasts dropped below the basal production levels of normal fibroblasts. Moreover, roscovitine caused a proportionately greater reduction in IL-6 production by SSc fibroblasts than by normal cells, suggesting that SSc fibroblasts may be more sensitive to the anti-fibrotic effects of roscovitine.

**Table 1:** Roscovitine decreases IL-6 secretion.

	*SSc supernatant*	*Normal supernatant*
Agent	IL-6 (pg/mL)	IL-6 (pg/mL)
DMSO vehicle	1129	196
Roscovitine 6.25 µg/mL	218	N.D.
Roscovitine 12.5 µg/mL	162	75

Results of ELISA analysis of fibroblast culture supernatants for IL-6, shown as pg/mL. Mean of duplicate determinations are shown. One representative experiment of two is shown. N.D., not determined.

## Discussion

Given the need for novel therapeutic approaches for SSc, we began to examine the efficacy of roscovitine in reducing the fibrotic phenotype of SSc fibroblasts. A selective inhibitor of CDK1, 2, 5, 7, and 9, roscovitine has been found to be antiproliferative in a wide range of cells. In normal human fibroblasts, roscovitine stops proliferation by inhibiting CDK2 [23]. Inhibition of cellular proliferation by roscovitine was proposed to underlie its effectiveness in suppressing matrix protein production in a mouse model of mesangial proliferative glomerulonephritis [1]. In our study, roscovitine decreased collagen and fibronectin mRNA and protein in normal and SSc fibroblasts that were growth arrested by confluence or by serum starvation without any further effect on the cell cycle (Figure 1). This indicates that the suppression of matrix protein production by roscovitine is a direct transcriptional effect rather than an epiphenomenon of growth arrest.

There is a growing body of literature delineating a role of CDKs and of CDK-inhibitors in modulating gene transcription (for review, see [14]). One can envision several pathways through which roscovitine inhibition of CDKs could regulate matrix protein production. CDK2 has several interactions that impact the transcription of matrix components. CDK2 is an activator of CBF/NF-Y binding [35], [36]. Binding of the CBF/NF-Y to CCAAT boxes activates transcription of multiple procollagen genes (including *COL1A1* and *COL2A1*) in response to TGF-β [37], [38], [39]. By inhibiting CDK2, in a p27-independent manner, roscovitine could decrease collagen transcription at the CBF/NF-Y binding site. Another mechanism through which roscovitine could inactivate multiple fibrotic genes would be inhibition of CDK2-p300 interactions. p300 has been shown to contribute to basal and to TGF-β-stimulated transcription of the collagen type I gene, acting in the latter instance through the SMAD transcriptional activator [40]. p300 also facilitates E2F-1 directed transcription of fibronectin in fibroblasts [41]. Downmodulation of fibronectin by roscovitine could result from its ability to inhibit CDK2/E2F/p300 complexes [42]. In addition, roscovitine could suppress expression of IL-6 by inhibiting CDK2-mediated activation of SP1 [43], a transcription factor that has been shown to be important in IL-6 gene expression [29], as well as in collagen expression [44], [45]. Whether CDK2 inhibition accounts for some of the effects of roscovitine is unclear, given that transduced p27 could not recapitulate roscovitine effects (Figure 1, Figure S2 in File S1). Roscovitine's functional effects are not limited to CDK2-inhibition—for instance, it has been noted elsewhere that ectopic p27 inhibited CDK2 but could not prevent SV40 from inducing quiescent fibroblasts to cycle, whereas roscovitine could prevent cycling [46].

We found that roscovitine enhanced both basal- and IL-6-induced STAT3 phosphorylation in fibroblasts (Figure 2). We have previously reported that roscovitine upregulated STAT3 DNA-binding and demonstrated that this could be mapped to its CDK2-inhibitory capability [47]. Robust IL-6/STAT3 signaling in roscovitine-exposed fibroblasts was somewhat surprising because roscovitine not only suppressed IL-6 secretion in both normal and SSc primary fibroblasts (Table 1), but also decreased the expression of fibronectin and CTGF, two proteins that were up-regulated by IL-6 (Figure 1).

Roscovitine may block IL-6 signaling distally by inhibiting CDK9. Inhibition of CDK9 can block the transcriptional elongation of STAT3-regulated transcripts by inactivating nuclear STAT3-CDK9 complexes. This mechanism has been shown to be involved in blockade of IL-6-mediated upregulation of fibrinogen by the CDK-inhibitor flavopiridol [48]. Although roscovitine did not block STAT3 phosphorylation by IL-6, it may have blocked STAT3 signal transduction in the nucleus by inhibiting CDK9-STAT3 complexes.

Although roscovitine did not block the ability of TGF-β to activate SMAD3, it may interfere with TGF-β signaling at a distal level. Phosphorylation of a SMAD linker region by CDK8 or 9 has been shown to augment both transcription and turnover [49]. Although that report demonstrated inhibition of SMAD1 linker phosphorylation by the broad CDK-inhibitor flavopiridol but not by roscovitine, this may reflect a necessity to inhibit both CDKs for full effect. A recent study showed that phosphorylation of polymerase β at Serine-2 is a critical step in TGF-β-mediated transcriptional activation [50]. This is the phosphorylation target of the CDK9-complex and can be regulated by roscovitine.

The suppression of matrix protein production by roscovitine was not specific to SSc fibroblasts. Collagen and fibronectin expression were suppressed both in primary fibroblasts from SSc patients, matched identical twin fibroblasts, control primary fibroblasts, and MRC-5 and 3T3 cell lines (Figures 1 and 3). It is likely that roscovitine downmodulates a common pathway required for matrix protein transcription under both normal and aberrant conditions rather than normalizing a signaling defect in extracellular matrix production that is aberrant in SSc. Functionally, the effect of roscovitine on SSc fibroblasts was profound, given that aberrantly high IL-6 levels in SSc fibroblasts were normalized by roscovitine (Table 1).

*CTGF* mRNA decreased in cells exposed to roscovitine despite ongoing production of TGF-β (an inducer of CTGF [51]) by the fibroblasts (Figures 1 and 3). CTGF itself contributes to TGF-β-mediated fibrosis, in part through promoting extracellular matrix production, and is also fibrogenic, independent of TGF-β. For example, while exogenous TGF-β on its own is insufficient to maintain fibrosis in murine models [52], [53], CTGF sustains fibrosis initiated by TGF-β in a mouse model [52]. By blocking production of CTGF, roscovitine can uncouple fibrosis from TGF-β in the microenvironment.

Cyclin-dependent kinases may provide attractive targets for new therapeutics in rheumatologic disease. While endogenous inhibitors of CDKs exhibit normal regulation in SSc (Figure S1 in File S1, as opposed to lupus [54] models), exogenous CDK-inhibitors such as roscovitine may prove to be useful therapeutic agents. An isomer of roscovitine (R-roscovitine, selciclib) has been widely used as an oral agent in phase II cancer clinical trials, following toxicity evaluation in phase I studies [55]. We used racemic roscovitine at concentrations (6.2 and 12.5 µg/mL) compatible with serum concentrations achieved in preclinical models [56], and furthermore detected effects on fibronectin expression at concentrations as low as 3 µg/mL (Figure S3 in File S1). Clinical trials use the R-isomer of roscovitine that is twice as potent as the S-isomer [16] at doses up to 2400 mg/day. At doses of 800 mg twice daily, serum concentrations of 2 µg/mL were achieved in Phase I anti-cancer trials [57]. Given that we used the less potent racemic roscovitine form, it is likely that anti-fibrotic activity could be achieved *in vivo*. Elaboration of the mechanism through which roscovitine suppresses fibrotic protein production could optimize the use of this class of agents for the treatment of SSc and possibly for use in other fibrotic diseases.

## Materials and Methods

### Cells and cell lines

Source of fibroblasts: Following the receipt of written informed consent, dermal fibroblasts were cultured from skin punch biopsies of three patients with diffuse cutaneous systemic sclerosis (dcSSc) and each patient's healthy identical twin. SSc patients met the American College of Rheumatology criteria for the diagnosis of SSc (Committee, 1980). Punch biopsies (6 mm) were performed at the leading edge of the lesion of each SSc patient and from a similar site in the healthy twin. Skin samples were minced and plated in 25-cm^2^ tissue culture flasks. Primary human fibroblasts were also cultured from the lung tissues of a normal donor whose lungs were not used for transplant surgery as previously described [58]. Archived specimens of these cells and of normal human dermal fibroblasts obtained from healthy donors were used for this study in conjunction with approval of the University of Pittsburgh IRB. Primary fibroblasts as well as MRC-5 (ATCC, Manassus, VA) and NIH/3T3 [47] cells were cultured in Dulbecco's modified Eagle's medium (Life Technologies) supplemented with 10% fetal bovine serum, 100 IU/mL penicillin, and 100 µg/mL streptomycin. Fibroblasts were used at passage numbers 4–10.

### Reagents

Fibronectin monoclonal antibody was obtained from NeoMarkers, Inc (Fremont, CA) or from Santa Cruz Biotechnology (Santa Cruz, CA). CTGF antibody from Torrey Pines (Secaucus, NJ) and collagen type I antibody was from Santa Cruz Biotechnology (Santa Cruz, CA). Phospho-Tyr705-STAT3, Phospho-p44/42-ERK1/2 and phospho-Ser 423/425-SMAD3 antibodies and corresponding antibodies to total STAT3, SMAD3 and ERK were from Cell Signaling Technology (Danvers, MA). Antibody against total and cleaved PARP was also from Cell Signaling Technology. Western blots were developed following incubation with HRP-conjugated secondary antibody using ECL kits (Amersham, GE Healthcare, Piscataway, NJ). Roscovitine was obtained from Calbiochem and was made up to 5 mg/mL in dimethylsulfoxide (DMSO), IL-6 was obtained from Peprotech (Rocky Hill, NJ) and stored at 5 µg/mL; AG490 was obtained from Calbiochem and was made up in DMSO to 25 mM. All reagents were stored in aliquots at −80°C.

### IL-6 determination

IL-6 levels were measured using a Quantikine assay kit (R & D Systems, Inc, Minneapolis, MN) according to manufacturer instructions. IL-6 levels in supernatants were measured using an EL312e Bio-Kinetics plate reader (Bio-Tek Instruments).

### Luciferase reporter assays

NIH/3T3 mouse fibroblast maintained in DMEM with 10% FBS were passaged to achieve 60% confluence 20 hours later. Cells were co-transfected with a COL2A1-promoter luciferase reporter plasmid (gracious gift from Mark E. Nuttall) and an eGFP-encoding plasmid (pEGFP-N1) using Polyfect lipid reagent (Qiagen, Inc., Valencia, CA) according to the manufacturer's instructions. Four hours post-transfection, medium was replaced either with roscovitine (6.25 ng/mL) or vehicle control. Twenty-four hours later, cells were harvested into lysis buffer (Promega, Inc., Madison, WI), frozen, thawed and assayed for luciferase activity. In parallel, aliquots were fractionated on SDS-PAGE gels, transferred and immunoblotted for GFP expression. Luciferase signal was normalized to GFP band intensity as measured using ImageJ software (ImageJ.nih.gov). Normalization to GFP was chosen after initial studies showed that a pTK-renilla transfection efficiency control was suppressed by roscovitine, consistent with another report [59].

### RNA preparation and Northern Blotting

RNA was prepared using the RNeasy kit (Qiagen, Valencia, CA) according to the manufacturer's instructions. Fifteen micrograms of RNA per sample was fractionated on a 1% agarose gel containing formaldehyde and MOPS, and Northern blotting was performed using standard capillary techniques. Membranes were hybridized with 10 million cpm/mL radiolabeled probe. The COL1A2-specific cDNA probe was generated by RT-PCR from normal fibroblast RNA using primers S: GCGGACTTTGTTGCTGCTTG and AS: AATGCCTTTGAAGCCAGGAAG.

### Immunocytochemical staining

Primary normal fibroblasts were cultured for 48 hours in the presence or absence of roscovitine. They were then washed in phosphate-buffered saline (PBS) containing 2% serum and 0.1% sodium azide, fixed for 10 minutes with cold acetone, and treated at room temperature with 0.2% Triton X-100 and 0.15 M HCl for 2 and 3 minutes, respectively. Following additional washing, the cells were blocked with 5% milk and incubated with anti-fibronectin antibody (5 µg/mL) for 30 minutes, followed by washing and secondary antibody for 30 minutes. Peroxide staining was performed using ABC kit (Vector laboratories) and DAB kit (Vector laboratories) according to manufacturer's instructions.

## Supporting Information

File S1 Supplemental Table and Figures.
(DOCX)
