# Supplementary material for: Antifibrotic Effects of Roscovitine in Normal and Scleroderma Fibroblasts
Source: PLoS One. 2012 Nov 20;7(11):e48560. doi: 10.1371/journal.pone.0048560 (PMC3502367; doi:10.1371/journal.pone.0048560)
Supplement: File S1 — Supplemental Table and Figures. (DOCX) [file pone.0048560.s001.docx]

| **TABLE S1. Quantitation of Western Blot Bands, by Figure** | | | | | | | | | | | | |
| --- | --- | --- | --- | --- | --- | --- | --- | --- | --- | --- | --- | --- |
| **Figure 1A** |  |  |  |  |  |  |  |  |  |  |  |  |
|  | Pellets | | | | | | | |  |  |  |  |
|  | SSc |  |  |  | NL |  |  |  |  |  |  |  |
|  | DMSO | 27 | Rosc | AG | DMSO | 27 | Rosc | AG |  |  |  |  |
| Fibronectin* | 46962 | 46819 | 34029 | 43996 | 44475 | 56908 | 24910 | 44857 |  |  |  |  |
| Collagen* | 6529 | 7016 | 696 | 4580 | 5254 | 5834 | 445 | 4725 |  |  |  |  |
| Loading | 1.0 | 0.9 | 0.9 | 1.0 | 1.1 | 0.9 | 0.8 | 1.0 |  |  |  |  |
|  |  |  |  |  |  |  |  |  |  |  |  |  |
|  | Supernatants | | | | | |  |  |  |  |  |  |
|  | SSc |  |  | NL |  |  |  |  |  |  |  |  |
|  | DMSO | Rosc | AG | DMSO | Rosc | AG |  |  |  |  |  |  |
| Fibronectin* | 12689 | 7218 | 14722 | 6340 | 3109 | 3461 |  |  |  |  |  |  |
| Collagen* | 868 | 194 | 821 | 445 | 272 | 423 |  |  |  |  |  |  |
| TGF-β* | 56834 | 62882 | 46697 | 47578 | 56114 | 39632 |  |  |  |  |  |  |
| Loading | 1.0 | 0.9 | 1.0 | 1.1 | 0.9 | 1.3 |  |  |  |  |  |  |
|  |  |  |  |  |  |  |  |  |  |  |  |  |
| **Figure 1C** |  |  |  |  |  |  |  |  |  |  |  |  |
|  | - rosc | + rosc |  |  |  |  |  |  |  |  |  |  |
| Collagen* | 5972 | 212 |  |  |  |  |  |  |  |  |  |  |
| Loading | 1.0 | 1.0 |  |  |  |  |  |  |  |  |  |  |
|  |  |  |  |  |  |  |  |  |  |  |  |  |
| **Figure 1D** |  |  |  |  |  |  |  |  |  |  |  |  |
|  | D | R | T | RT | 6 | R6 |  |  |  |  |  |  |
| Fibronectin* | 9390 | 2615 | 7240 | 6225 | 14990 | 5181 |  |  |  |  |  |  |
| Collagen* | 11809 | 118 | 10302 | 73 | 5937 | 0 |  |  |  |  |  |  |
| CTGF* | 1302 | 1097 | 17182 | 7889 | 5918 | 1410 |  |  |  |  |  |  |
| PARP* | 9286 | 8526 | 9523 | 8101 | 8537 | 10262 |  |  |  |  |  |  |
| Loading | 1.0 | 1.0 | 1.0 | 0.9 | 0.9 | 0.8 |  |  |  |  |  |  |
|  |  |  |  |  |  |  |  |  |  |  |  |  |
| **Figure 1E** |  |  |  |  |  |  |  |  |  |  |  |  |
|  | NL |  |  |  |  |  | SSc |  |  |  |  |  |
|  | D | R | T | RT | 6 | R6 | D | R | T | RT | 6 | R6 |
| Fibronectin* | 29527 | 17880 | 27840 | 16753 | 27769 | 20688 | 12535 | 7735 | 26720 | 18568 | 21843 | 10595 |
| Collagen* | 22392 | 5476 | 22540 | 5357 | 26214 | 7343 | 9917 | 4738 | 18531 | 1994 | 14683 | 3581 |
| CTGF* | 2801 | 2773 | 12570 | 2817 | 968 | 2903 | 1754 | 1261 | 11447 | 5243 | 6763 | 857 |
| Loading | 1.0 | 1.0 | 1.0 | 0.9 | 0.8 | 0.9 | 1.0 | 1.1 | 0.8 | 0.9 | 1.0 | 1.0 |
|  |  |  |  |  |  |  |  |  |  |  |  |  |
|  |  |  |  |  |  |  |  |  |  |  |  |  |
|  |  |  |  |  |  |  |  |  |  |  |  |  |
|  |  |  |  |  |  |  |  |  |  |  |  |  |
|  |  |  |  |  |  |  |  |  |  |  |  |  |
| *Corrected for loading |  |  |  |  |  |  |  |  |  |  |  |  |
|  |  |  |  |  |  |  |  |  |  |  |  |  |
|  |  |  |  |  |  |  |  |  |  |  |  |  |
|  |  |  |  |  |  |  |  |  |  |  |  |  |
|  |  |  |  |  |  |  |  |  |  |  |  |  |
|  |  |  |  |  |  |  |  |  |  |  |  |  |
| **Figure 2A** |  |  |  |  |  |  |  |  |  |  |  |  |
|  | MRC-5 |  |  |  |  |  |  |  |  |  |  |  |
|  | D | R | T | RT | 6 | R6 |  |  |  |  |  |  |
| pSTAT3^#^ | 390 | 1660 | 394 | 5316 | 8644 | 11905 |  |  |  |  |  |  |
| STAT3* | 10010 | 10657 | 10779 | 11698 | 11153 | 12471 |  |  |  |  |  |  |
| pSMAD3^#^ |  |  | 0.49 | 0.26 |  |  |  |  |  |  |  |  |
| SMAD3* | 10563 | 11408 | 17073 | 16845 | 9439 | 11201 |  |  |  |  |  |  |
| pERK* | 20656 | 28683 | 29092 | 31578 | 29974 | 29117 |  |  |  |  |  |  |
| Loading | 1.0 | 0.9 | 0.9 | 0.8 | 0.9 | 0.9 |  |  |  |  |  |  |
|  |  |  |  |  |  |  |  |  |  |  |  |  |
|  | NL57 |  |  |  |  |  |  |  |  |  |  |  |
|  | D | R | T | RT | 6 | R6 |  |  |  |  |  |  |
| pSTAT3^#^ |  |  |  | 0.01 | 0.14 | 0.47 |  |  |  |  |  |  |
| STAT3* | 6302 | 7046 | 7870 | 7501 | 7355 | 7050 |  |  |  |  |  |  |
| pSMAD3^#^ |  |  | 0.37 | 0.21 |  |  |  |  |  |  |  |  |
| SMAD3* | 6802 | 7418 | 8430 | 7424 | 8577 | 8325 |  |  |  |  |  |  |
| pERK* | 2940 | 14291 | 7357 | 13917 | 11939 | 10772 |  |  |  |  |  |  |
| Loading | 1.0 | 1.0 | 1.0 | 0.9 | 0.9 | 0.9 |  |  |  |  |  |  |
|  |  |  |  |  |  |  |  |  |  |  |  |  |
|  | NL |  |  |  |  |  | SSc |  |  |  |  |  |
|  | D | R | T | RT | 6 | R6 | D | R | T | RT | 6 | R6 |
| pSTAT3^#^ |  |  |  |  | 0.17 | 0.36 |  |  |  |  | 0.12 | 0.30 |
| STAT3* | 19024 | 16445 | 18722 | 18425 | 17862 | 17866 | 15487 | 17038 | 13630 | 13684 | 14705 | 21445 |
| pSMAD3^#^ |  |  | 0.49 | 0.59 |  |  |  |  | 1.46 | 1.27 |  |  |
| SMAD3* | 6828 | 7717 | 12154 | 9935 | 7578 | 7640 | 2566 | 2396 | 2476 | 2761 | 2123 | 1842 |
| pERK* | 6312 | 7526 | 7078 | 6861 | 5054 | 7323 | 7999 | 9861 | 11892 | 13883 | 11025 | 13294 |
| Loading | 1.0 | 1.1 | 1.0 | 1.1 | 1.1 | 0.9 | 1.0 | 1.1 | 1.0 | 1.0 | 1.0 | 1.0 |
|  |  |  |  |  |  |  |  |  |  |  |  |  |
| **Figure 2B** |  |  |  |  |  |  |  |  |  |  |  |  |
|  | 0 | 0 | 30 | 30 | 60 | 60 | 0 | 60 | 60 |  |  |  |
|  | - | + | - | + | - | + | - |  | + |  |  |  |
| pSMAD3^#^ |  |  | 0.37 | 0.323 | 0.232 | 0.411 |  | 0.745 | 0.658 |  |  |  |
| SMAD3* | 14429 | 9084 | 25103 | 25561 | 21346 | 19415 | 14638 | 13573 | 18433 |  |  |  |
| Loading | 1.0 | 1.0 | 0.9 | 0.9 | 1.0 | 1.0 | 1.0 | 1.1 | 1.1 |  |  |  |

*Corrected for loading

^#^Compared to total protein

Densitometry was performed of all bands in Figures 1 and 2. Protein loading was quantitated and normalized to values for the control (DMSO vehicle) condition. Densitometric readings of collagen, fibronectin, CTGF and PARP bands were normalized to the normalized protein loading for each sample. Phospho-SMAD and phospho-STAT signals were normalized to total SMAD or STAT protein values, respectively.

**Supplemental Figures**

**
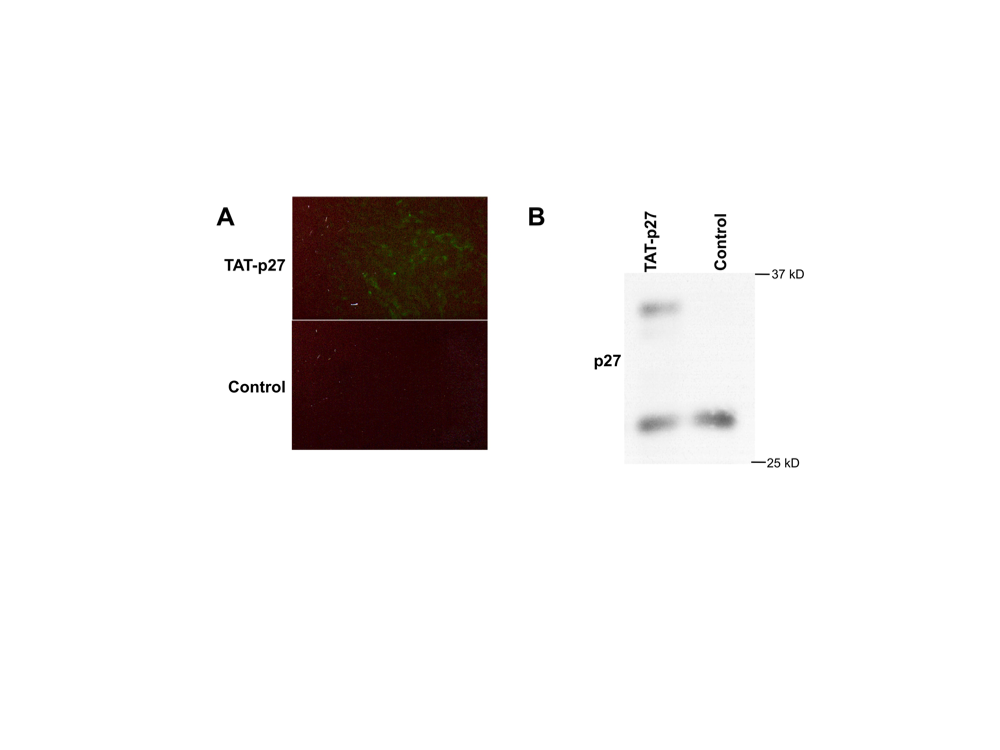
**

**Figure S1.** **p27-TAT transduction.** Direct transduction into scleroderma fibroblasts of 150 nM

FITC-labeled p27 protein fused to a (membrane-permeant) 11 amino acid TAT peptide tag is shown.

Transduced cells are FITC-positive; full-length TAT-p27 fusion protein in addition to endogenous p27

is evident upon Western blotting of these cell extracts.


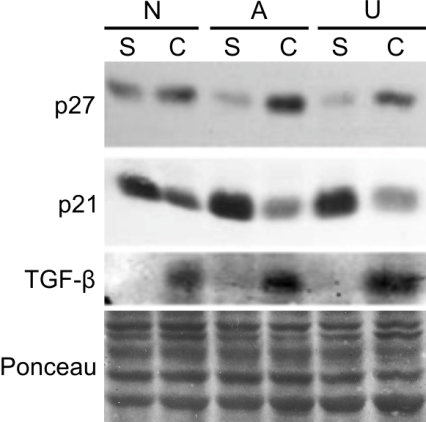


**Figure S2. Confluence-dependent expression of CDK-inhibitors in scleroderma and**

**normal fibroblasts.** Passage 4 fibroblasts cultured from affected (A) or unaffected (U) skin of

a scleroderma patient and normal twin (N) were harvested at subconfluent (S) and confluent (C)

cell densities as shown. Cells were cultured in DMEM containing 10% serum. 50 μg of protein extract

was analyzed for the expression of cdk-inhibitors p21 and p27, and for the expression of TGF-β.

Similar responses were seen in another independent set of scleroderma fibroblasts (data not shown).

**
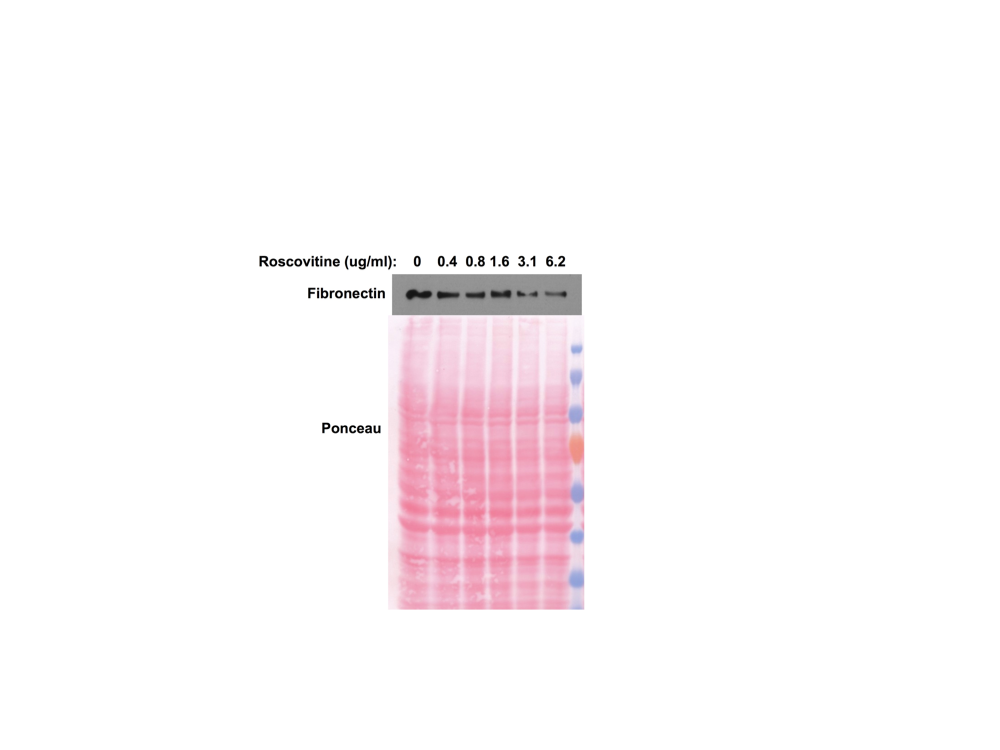
**

**Figure S3. Roscovitine titration.** Decreased expression of fibronectin by roscovitine-exposed

fibroblasts was evident at roscovitine values as low as 3 μg/mL. Equal loading is shown by Ponceau staining.
